# A Pauci-Immune Synovial Pathotype Predicts Inadequate Response to TNFα-Blockade in Rheumatoid Arthritis Patients

**DOI:** 10.3389/fimmu.2020.00845

**Published:** 2020-05-05

**Authors:** Alessandra Nerviani, Maria Di Cicco, Arti Mahto, Gloria Lliso-Ribera, Felice Rivellese, Georgina Thorborn, Rebecca Hands, Mattia Bellan, Daniele Mauro, Marie-Astrid Boutet, Giovanni Giorli, Myles Lewis, Stephen Kelly, Michele Bombardieri, Frances Humby, Costantino Pitzalis

**Affiliations:** Experimental Medicine and Rheumatology, William Harvey Research Institute, Queen Mary University of London, London, United Kingdom

**Keywords:** synovial tissue, anti-TNF, pathotype, rheumatoid arthritis, certolizumab-pegol

## Abstract

**Objectives:** To assess whether the histopathological features of the synovium before starting treatment with the TNFi certolizumab-pegol could predict clinical outcome and examine the modulation of histopathology by treatment.

**Methods:** Thirty-seven RA patients fulfilling UK NICE guidelines for biologic therapy were enrolled at Barts Health NHS trust and underwent synovial sampling of an actively inflamed joint using ultrasound-guided needle biopsy before commencing certolizumab-pegol and after 12-weeks. At 12-weeks, patients were categorized as responders if they had a DAS28 fall >1.2. A minimum of 6 samples was collected for histological analysis. Based on H&E and immunohistochemistry (IHC) staining for CD3 (T cells), CD20 (B cells), CD138 (plasma cells), and CD68 (macrophages) patients were categorized into three distinct synovial pathotypes (lympho-myeloid, diffuse-myeloid, and pauci-immune).

**Results:** At baseline, as per inclusion criteria, DAS28 mean was 6.4 ± 0.9. 94.6% of the synovial tissue was retrieved from the wrist or a metacarpophalangeal joint. Histological pathotypes were distributed as follows: 58% lympho-myeloid, 19.4% diffuse-myeloid, and 22.6% pauci-immune. Patients with a pauci-immune pathotype had lower levels of CRP but higher VAS fatigue compared to lympho- and diffuse-myeloid. Based on DAS28 fall >1.2, 67.6% of patients were deemed as responders and 32.4% as non-responders. However, by categorizing patients according to the baseline synovial pathotype, we demonstrated that a significantly higher number of patients with a lympho-myeloid and diffuse-myeloid pathotype in comparison with pauci-immune pathotype [83.3% (15/18), 83.3 % (5/6) vs. 28.6% (2/7), *p* = 0.022) achieved clinical response to certolizumab-pegol. Furthermore, we observed a significantly higher level of post-treatment tender joint count and VAS scores for pain, fatigue and global health in pauci-immune in comparison with lympho- and diffuse-myeloid patients but no differences in the number of swollen joints, ESR and CRP. Finally, we confirmed a significant fall in the number of CD68+ sublining macrophages post-treatment in responders and a correlation between the reduction in the CD20+ B-cells score and the improvement in the DAS28 at 12-weeks.

**Conclusions:** The analysis of the synovial histopathology may be a helpful tool to identify among clinically indistinguishable patients those with lower probability of response to TNFα-blockade.

## Introduction

Rheumatoid Arthritis (RA) is a chronic inflammatory autoimmune disease characterized by persistent inflammation of synovial tissue. If not adequately treated, it can lead to progressive structural damage and subsequent disability (1). Early diagnosis and the introduction of advanced therapeutics for RA patients has resulted in substantial improvements in clinical outcomes. However, irrespective of drug choice or therapeutic target, response rates have remained stubbornly fixed with approximately 60% of patients attaining a modest 20% improvement in disease activity (ACR20), while only around 20% of patients achieving remission (2). In addition, the mechanisms responsible for such variable response to therapy are still unknown, and there are no biomarkers in regular clinical use which are predictive of treatment response to individual therapeutic agents (3). This is reflected in the most recent EULAR recommendations for the management of RA (4) that support the use of any of the available targeted therapeutics following failure on conventional synthetic (cs) DMARDs, implying a “trial and error” approach to treatment. Such an approach leaves a huge unmet need and exposes patients to a therapeutic lottery of drugs, which they may not respond to and which may cause unnecessary adverse effects. Thus, there has been considerable interest in trying to identify biomarkers of treatment response in the diseased tissue (5), as the search in peripheral blood has been largely unsuccessful (3). Heterogeneity in the quantity and quality of synovial cellular infiltrate is well recognized, and clustering of patients into three histological categories (pathotypes) has recently been described in a large early RA cohort as (i) *lympho-myeloid*, characterized by well-organized B or plasma cell aggregates and rich in macrophages; (ii) *diffuse-myeloid*, with predominant macrophages within the sublining tissue and lacking B/plasma cell aggregates, and (iii) *pauci-immune*, with scant infiltration of immune cells and prevalence of resident fibroblasts (6). Importantly, synovial molecular signatures have also been identified to associate with each pathotype (7). Moreover, synovial pathotypes and molecular signatures in the baseline biopsy are associated with clinical phenotypes, clinical response to csDMARDs, radiologic progression 12 months after the original biopsy (6, 7), and predict future requirement for biologic therapy (8). These data suggest that synovial biomarkers may be useful tools to stratify patients' response to biologic therapy. TNF inhibitors (TNFi) are the most widely prescribed biologic drugs in RA, and thus biomarkers of responses have been the most intensively sought. However, whilst levels of TNFα in the peripheral blood have repeatedly and consistently proven to be ineffective predictors (9), synovial TNFα expression and factors such as the presence of synovial B cell aggregates or specific gene signatures have been variably associated with clinical outcomes (10–12).

In this study, therefore, capitalizing on the current understanding of rheumatoid synovial histopathology, we aimed to investigate whether specific histological features in synovium prior to starting treatment with the TNFi certolizumab-pegol could predict clinical outcome, and furthermore we examined the modulation of histopathology by treatment.

## Methods

### Patients

Thirty-seven RA patients [using the 1987 revised American College of Rheumatology (ACR) classification criteria] (13) fulfilling UK National Institute for Health and Care Excellence (NICE) prescribing guidelines for biologic therapy (i.e., failure of at least two csDMARDs and highly active disease demonstrated by DAS28 > 5.1) (14) were enrolled in the study at Barts Health NHS trust. Patients were excluded from the study if daily oral prednisolone dose exceeded 10 mg or if they have previously received any biologic treatment, including non-anti-TNF agents. Routine demographics including age, seropositivity for rheumatoid factor and anti-citrullinated peptide antibodies, disease duration, concomitant therapy and disease activity assessment (DAS28) were performed at baseline. Patients underwent synovial sampling of an actively inflamed joint using ultrasound (US)—guided needle biopsy as previously described (15) with collection of pre-biopsy US images. US images of the biopsied joints were scored for both synovial thickening (ST) and Power-Doppler (PD) using the EULAR/OMERACT US semi-quantitative synovitis score system (0–3) (16). Patients were subsequently commenced on standard treatment with subcutaneous certolizumab-pegol (400 mg at 0–2–4-weeks, then 200 mg every other week). Twelve-weeks after starting treatment, the number of swollen and tender joints (28 joint count), patient visual analog score (VAS) for pain, fatigue, global health and physician assessment of global health were recorded along with Health Assessment Questionnaire (HAQ). Patients were categorized as responders to therapy if a DAS28 fall >1.2 was demonstrated between baseline and 12-weeks, and non-responders if it did not. At 12-weeks, patients were consented to undergo a second synovial biopsy of the same joint sampled as at baseline. The study was approved by the local ethics committee (Rec. No. 10/H0801/47) and written informed consent was obtained from all patients prior to inclusion.

### Histological Assessment of the Synovial Tissue

A minimum of 6 samples was retrieved for histological analysis from each biopsy procedure for subsequent paraffin embedding. Following H&E staining of 3 μm-cut paraffin-embedded sections, synovial tissue underwent immunohistochemical (IHC) staining for CD3 (T cells), CD20 (B cells), CD138 (plasma cells), and CD68 (macrophages), as previously described (17). Synovial tissue was deemed as gradable if there was a visible lining layer and/or presence of macrophages in the sublining. Two independent scorers expert in synovial tissue histology quantified the degree of the immune infiltrate using a semi-quantitative score (0–4) as previously described (17). Subsequently, synovial samples were categorized into three distinct synovial pathotypes based on the following criteria: (i) Lympho-myeloid, if CD20^+^ B-cells score ≥ 2 and/or CD138^+^ plasma cells score > 2; (ii) Diffuse-myeloid, if CD68^+^ sublining (SL) macrophages score ≥ 2, CD20^+^ B-cells score ≤ 1 and CD138^+^ plasma cells score ≤ 2; Pauci-immune, if CD68SL macrophages score < 2 and CD3^+^ T cells score, CD20^+^ B cells score, and CD138^+^ plasma cells score < 1 (6).

### Statistical Analysis

Statistical analyses were performed using GraphPad Prism version 8.2.1. A *p* < 0.05 was considered statistically significant. Differences in continuous variables between two groups were analyzed by T-test or Mann-Whitney U-test depending on normality. Differences in variables between three or more groups were assessed through one-way ANOVA or Kruskal-Wallis with Dunn's correction test. Wilcoxon matched-pairs rank test was used to compare matched samples (e.g., pre- and post-treatment variables in the same patient). Chi-squared or Fisher's exact test was applied to analyze the significance of the association between categorical variables. Spearman's correlation test was used to assess the presence of significant correlations between variables. Multiple logistic regression analysis was performed with GraphPad Prism version 8.3.1. The binary clinical response (based on DAS28 improvement ≥1.2) was used as the outcome. The primary model was defined by the main effect of the pathotype only. Additional models were adjusted by the inclusion of several covariates such as age, gender, RF/CCP status and baseline DAS28. The Sankey diagram in **Figure 5** was plotted using SankeyMATIC (http://sankeymatic.com).

## Results

### Patients' Characteristics

Patients' baseline demographic and clinical features are summarized in Table 1. Briefly, as expected in a population of established RA, ~80% of patients were female, and the average age was 51.3 ± 11.7 years. About 70% of patients were either rheumatoid factor (RF) or anti-cyclic citrullinated peptide (CCP) antibody positive. As per the inclusion criteria of the study, all patients had high disease activity (DAS28 6.4 ± 0.9). All patients were previously exposed to csDMARDs treatment but were naïve to any biologics, and 35.1% of patients were on concomitant steroid treatment (≤ 10 mg per day) at the time of the recruitment.

**Table 1:** Baseline characteristics of the population included in the study (*n* = 37).

Female % (*n*)		81% (30)
Age years (mean ± *SD*)		51.3 ± 11.7
Disease duration years (mean ± *SD*)		6.3 ± 5.8
Smoking status	Previous % (*n*)	38.9% (14)
	Current % (*n*)	22.2% (8)
RF+ % (*n*)		64.9% (24)
Anti-CCP+ % (*n*)		64.9% (24)
RF+ or Anti-CCP+ % (*n*)		70.3% (26)
ESR mm/h (mean ± *SD*)		31.1 ± 25.8
CRP mg/l mean (mean ± *SD*)		10.7 ± 20.2
TJ/28 (mean ± *SD*)		18 ± 8
SJ/28 (mean ± *SD*)		10 ± 4
VAS GH patient (mean ± *SD*)		82.1 ± 13.9
VAS GH physician (mean ± *SD*)		73.7 ± 15.2
VAS pain (mean ± *SD*)		67.8 ± 24.9
VAS tiredness (mean ± *SD*)		55.1 ± 25.2
HAQ (mean ± *SD*)		1.7 ± 0.7
DAS28 (mean ± *SD*)		6.4 ± 0.9
DMARDs	MTX % (*n*)	24.3% (9)
	LFN % (*n*)	5.4% (2)
	MTX+HCQ % (*n*)	43.3% (16)
	MTX+SSZ % (*n*)	24.3% (9)
	MTX+SSZ+HCQ % (*n*)	2.7% (1)
Steroid treatment % (*n*)		35.1% (13)
Biopsied joint	MCP % (*n*)	21.6% (8)
	Elbow % (*n*)	2.7% (1)
	Knee % (*n*)	2.7% (1)
	Wrist % (*n*)	73% (27)
US BJ	Synovial Thickening (mean ± *SD*)	2.4 ± 0.6
	Power Doppler (mean ± *SD*)	1.6 ± 1

*SD, Standard Deviation; n, number; RF, Rheumatoid Factor; CCP, Cyclic Citrullinated Peptide; ESR, erythrocyte sedimentation rate; CRP, C-Reactive Protein; TJ, Tender Joints; SJ, Swollen Joints; VAS, Visual Analog Scale (0-100); GH, Global Health; HAQ, Health Assessment Questionnaire; DAS, Disease Activity Score; DMARDs, Disease Modifying Anti-Rheumatic Drugs; MTX, methotrexate; LFN, leflunomide; HCQ, hydroxychloroquine; SSZ, sulfasalazine; MCP, metacarpophalangeal; US BJ, Ultrasound Biopsied Joint*.

### Histological Classification

**Figure 1:**
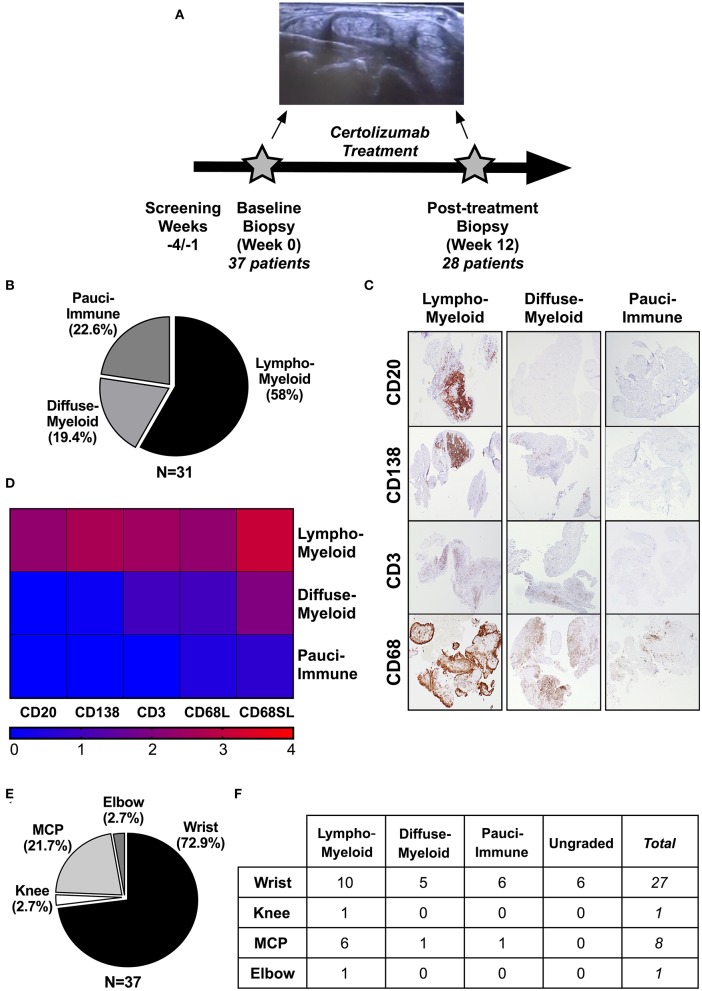
**(A–F)** Design of the study and histological characterization of the synovial tissue. **(A)** Timeline of the study, which included a baseline US-guided needle synovial biopsy at week 0 (37 patients) and a second biopsy of the same joint after 12-weeks of treatment with certolizumab-pegol (28 patients). At the top, representative gray-scale transverse section of an US-guided wrist biopsy showing the needle entering the joint space underneath the IV extensor tendons compartment. **(B)** Distribution (%) of synovial pathotypes at baseline. **(C)** Representative images of immunohistochemistry staining of synovial tissue for immune cells markers and classification in three pathotypes: *lympho-myeloid, diffuse-myeloid* and *pauci-immune*. Original magnification 4x. **(D)** Heatmap showing the degree of infiltration of immune cells (CD20, B-cells; CD138, plasma cells; CD3, T-cells; CD68L, macrophages of the lining; CD68SL, macrophages of the sublining) in each pathotype. **(E)** Distribution (%) of biopsied joints. **(F)** Histological pathotype according to synovial biopsy site. MCP, metacarpophalangeal. Fisher's test: not significant.

A total of 37 patients were recruited to the study and underwent a synovial biopsy at study entry (baseline). 28/37 patients subsequently consented to a second synovial biopsy at 12-weeks follow up (Figure 1A, Supplementary Figures 1, 2). 31/37 baseline synovial biopsies and 22/28 12-weeks repeated biopsies yielded synovial tissue of sufficient quality for subsequent histological analysis. Demographics and clinical features of patients classified as “ungraded” (6 patients at baseline and 6 patients at 12-weeks) as well as those of patients who did not undergo a repeated synovial biopsy post-treatment are summarized in Supplementary Tables 1, 2. At baseline, 58% (18/31) were classified as lympho-myeloid, 19.4% (6/31) as diffuse-myeloid, and 22.6% (7/31) as pauci-immune (Figure 1B). Representative immunohistological images are shown in Figure 1C, and the relative degree of immune cell infiltrate per pathotype shown in Figure 1D. 94.6% of the synovial tissue was retrieved from the wrist or a metacarpophalangeal joint (Figure 1E). There was no difference in the pathotype distribution among the various biopsy sites (Figure 1F).

### Synovial Pathotypes and Baseline Clinical Features

**Figure 2:**
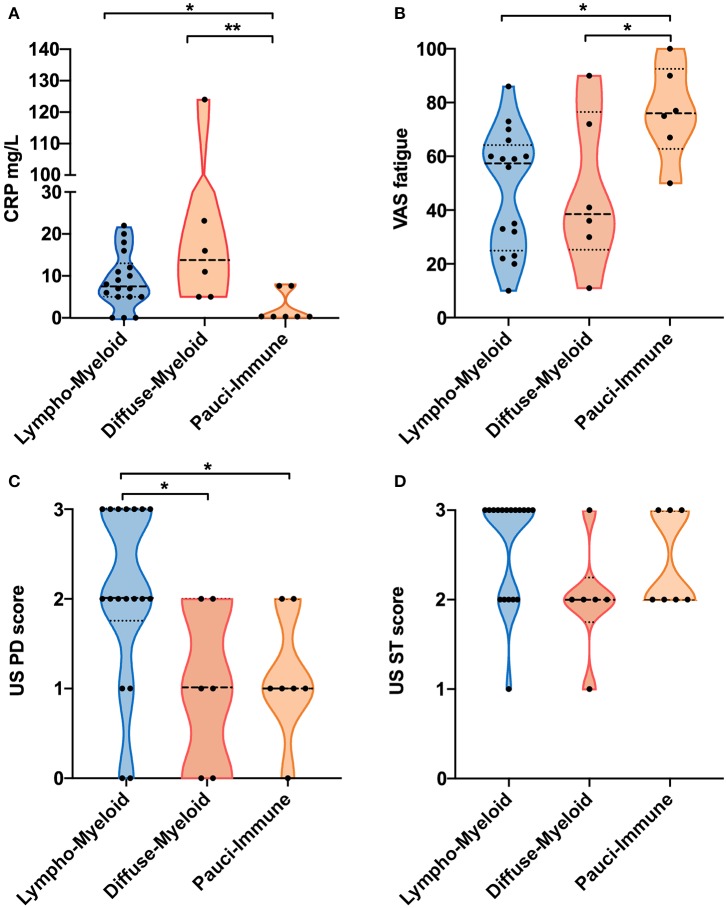
**(A–D)** Violin plots showing differences in CRP **(A)**, VAS fatigue (0–100) **(B)**, ultrasound (US) Power-Doppler (PD) score (0–3) **(C)** and US synovial thickening (ST) score measured in gray-scale (0–3) **(D)** of the biopsied joints between pathotype groups. Median and interquartile ranges are represented by thick and thin dotted lines, respectively. ^**^p < 0.01, ^*^p < 0.05, Kruskal-Wallis with *post-hoc* multiple comparison on 31 patients.

We next evaluated whether there were significant differences in clinical parameters between patients stratified according to baseline synovial pathotype (Table 2). We demonstrated significantly lower levels of C-Reactive Protein (CRP) (Figure 2A) but higher VAS fatigue score (Figure 2B) in patients with a pauci-immune compared to lympho- and diffuse-myeloid pathotypes. The US Power Doppler (USPD) score of the biopsied joint was significantly higher in lympho-myeloid patients, while the US synovial thickening (USST), measured on gray-scale, was comparable among the three pathotypes (Figures 2C,D). This suggests that although the total proliferative cellular burden is the same across the pathotypes there are differences in the nature of the inflammatory milieu between the pathotypes, with increased vascularity observed in the lympho-myeloid pathotype.

**Table 2:** Analysis of baseline features by pathotype.

		**Lymphoid-Myeloid (58%, *n* = 18)**	**Diffuse-Myeloid (19.4%, *n* = 6)**	**Pauci-Immune (22.6%, *n* = 7)**	***p-value***
Female % (*n*)		77.8% (14)	100% (6)	100% (7)	0.356^a^
Age years (mean ± *SD*)		50.4 ± 10.8	47.5 ± 17.6	54.7 ± 11.8	0.577^b^
Disease duration years (mean ± *SD*)		5.05 ± 3.7	4.8 ± 5.3	5.1 ± 6.3	0.992^b^
RF+ % (*n*)		72.2% (13)	50% (2)	71.4% (5)	0.588^a^
ACPA + % (*n*)		72.2% (13)	50% (3)	57.1% (4)	0.595^a^
RF or ACPA + % (*n*)		77.8% (14)	50% (3)	71.4% (5)	0.418^a^
ESR mm/h (mean ± *SD*)		30.53 ± 24.74	54.5 ± 33.2	25.4 ± 21.2	0.107^b^
ESR mm/h (mean ± *SD*)		30.53 ± 24.74	54.5 ± 33.2	25.4 ± 21.2	0.107^b^
CRP mg/l (mean ± *SD*)		8.9 ± 6.6	30.6 ± 46.2	2.2 ± 3.9	^*^0.020^b^
TJ/28 (mean ± *SD*)		17.4 ± 8.7	13 ± 6.4	22.6 ± 4.3	0.087^b^
SJ/28 (mean ± *SD*)		10.5 ± 5	8.7 ± 2.7	9.3 ± 4.9	0.675^b^
VAS GH pt (mean ± *SD*)		83.5 ± 13.6	72.7 ± 16.4	86.3 ± 16.3	0.230^b^
VAS GH phys (mean ± *SD*)		79.9 ± 12.6	65.2 ± 22	71 ± 12.5	0.112^b^
VAS pain (mean ± *SD*)		63.9 ± 27.9	54.7 ± 25.2	87.2 ± 16.6	0.101^b^
VAS tiredness (mean ± *SD*)		47.8 ± 22.6	46.7 ± 29	76.5 ± 17.5	^*^0.038^b^
HAQ (mean ± *SD*)		1.7 ± 0.7	1.4 ± 0.8	2 ± 0.8	0.451^b^
DAS28 (mean ± *SD*)		6.4 ± 1	6.5 ± 0.8	6.7 ± 1	0.787^b^
Steroid % (n)		27.8% (5)	50% (3)	42.9% (3)	0.595^a^
USST biopsied joint		2.6 ± 0.6	2 ± 0.6	2.4 ± 0.5	0.112^b^
USPD biopsied joint		2.1 ± 1	1 ± 0.9	1.1 ± 0.7	^*^0.023^b^
Steroid treatment % (*n*)		27.8% (5)	50% (3)	42.8 (3)	0.595^a^
DMARDs	MTX % (*n*)	33.3% (6)	0% (0)	14.3% (1)	^c^>0.999
	LFN % (*n*)	5.55% (1)	0% (0)	14.3% (1)	
	MTX+HCQ % (*n*)	27.8% (5)	66.7% (4)	57.1% (4)	
	MTX+SSZ % (*n*)	27.8% (5)	33.3% (2)	14.3% (1)	
	MTX+SSZ+HCQ % (*n*)	5.55% (1)	0% (0)	0% (0)	

Baseline characteristics were compared by Fisher's exact test^a^ or Kruskal-Wallis with post-hoc Dunn's test^b^ as appropriate. ^c^DMARDs treatment distribution was analyzed as monotherapy vs. multiple DMARDs in lympho/diffuse-myeloid vs. pauci-immune. ^*^p < 0.05 was considered statistically significant. SD, Standard Deviation; n, number; RF, Rheumatoid Factor; CCP, Cyclic Citrullinated Peptide; ESR, erythrocyte sedimentation rate; CRP, C-Reactive Protein; TJ, Tender Joints; SJ, Swollen Joints; VAS, Visual Analog Scale (0–100); GH, Global Health; HAQ, Health Assessment Questionnaire; DAS, Disease Activity Score; DMARDs, Disease Modifying Anti-Rheumatic Drugs; MTX, methotrexate; LFN, leflunomide; HCQ, hydroxychloroquine; SSZ, sulfasalazine; MCP, metacarpophalangeal; USST, Ultrasound Synovial Thickening; USPD, Ultrasound Power-Doppler.

### Baseline Synovial Histological Pathotypes Associate With 12-Weeks Response to Certolizumab-Pegol

**Figure 3:**
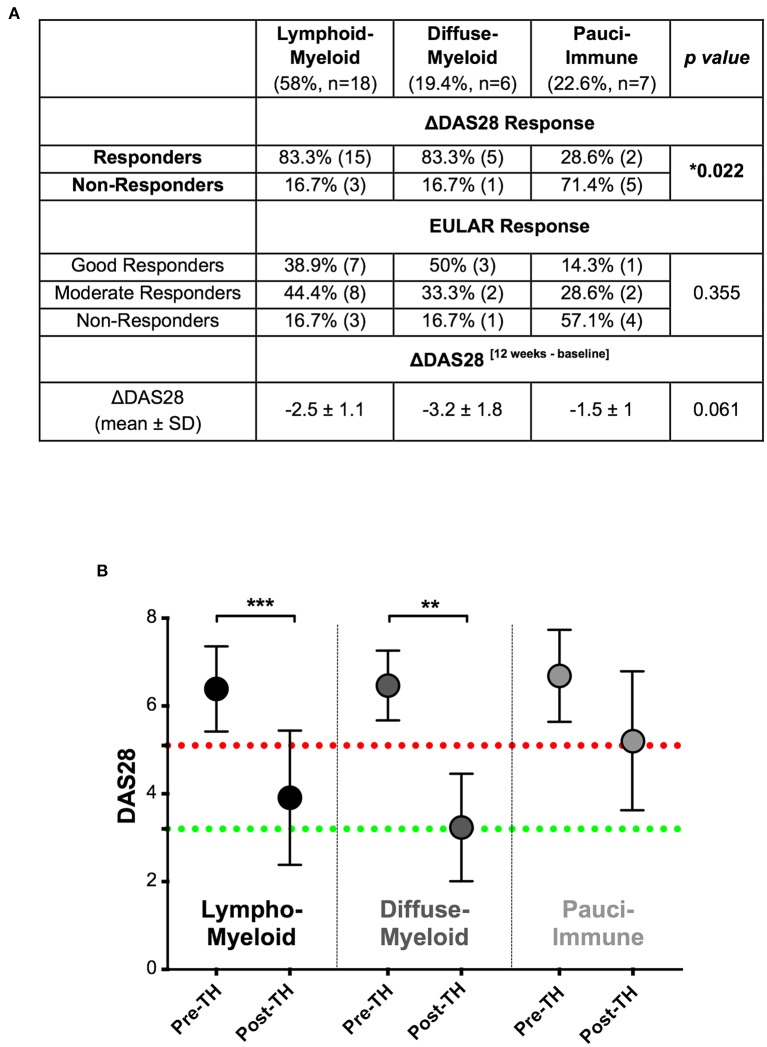
**(A)** Table summarizing clinical response rates by pathotypes to certolizumab-pegol at 12-weeks. ΔDAS28 was calculated by subtracting the baseline-DAS28 value from the 12-weeks-DAS28; DAS28 fall > 1.2 defined “responders.” Distribution of response rates was tested by Fisher's exact test while differences in ΔDAS28 by Kruskal-Wallis with Dunn's test. SD, standard deviation. **(B)** Comparison of pre- (pre-TH) and post-treatment (post-TH) DAS28 by pathotype. ^***^p < 0.001, ^**^p < 0.01, Kruskal-Wallis with *post-hoc* Dunn's multiple comparison test on 31 patients. Red dotted line represents DAS28 5.1 (“high disease activity”); green dotted line represents DAS28 3.2 (“low disease activity”).

Twelve-weeks after commencing certolizumab-pegol, 25/37 patients (67.6%) were classified as responders and 12/37 (32.4%) as non-responders based on a DAS28 fall >1.2 (ΔDAS28 response). We next stratified patients according to synovial pathotype and evaluated whether there were significant differences in clinical outcomes between groups. We demonstrated that a significantly higher number of patients with a lympho-myeloid and diffuse-myeloid pathotype in comparison with pauci-immune pathotype [83.3% (15/18), 83.3 % (5/6) vs. 28.6% (2/7), Fisher test *p* = 0.022] were classified as responders to therapy. A similar distribution was observed when EULAR response criteria were applied: in this case, the rate of EULAR non-responders was 16.7% in both lympho-myeloid and diffuse-myeloid in comparison to 57.1% in pauci-immune patients (Figure 3A). Consistent with this, we also observed a significant fall in DAS28 score pre- and post-treatment in both the lympho-myeloid and the diffuse-myeloid groups [6.4 ± 1 to 3.9 ± 1.5 (*p* < 0.001) and 6.5 ± 0.8 to 3.2 ± 1.2 (*p* = 0.002) respectively] but not in the pauci-immune group [6.7 ± 1 to 5.2 ± 1.6 (*p* = 0.06)] (Figure 3B). Using a dichotomic classification of the pathotypes (lympho-myeloid and diffuse-myeloid vs. pauci-immune) we observed a significantly lower response in the pauci-immune group (Fisher test *p* = 0.01). The sensitivity of this test as a predictor of response was 83% with a specificity of 71%; the positive predictive value was 90% and the negative predictive value 56%. A logistic regression model including the pathotype alone mirrored the results of the Fisher test (“Model 1” in Supplementary Table 3). The histological classification remained significant when the model was adjusted for age, gender and RF/CCP status (“Model 2” in Supplementary Figure 2) as well as in a larger model including also DAS28 at baseline (“Model 3” in Supplementary Figure 2).

### An Inadequate Response to Certolizumab-Pegol in Patients Categorized as Pauci-Immune Pathotype Is Driven by Higher Levels of Tender Joint Counts and Patient-Reported-Outcomes

As patients with a baseline pauci-immune histological pattern were less likely to respond to certolizumab-pegol, we next evaluated differences in individual components of clinical response between pathotype groups at 12-weeks.

**Figure 4:**
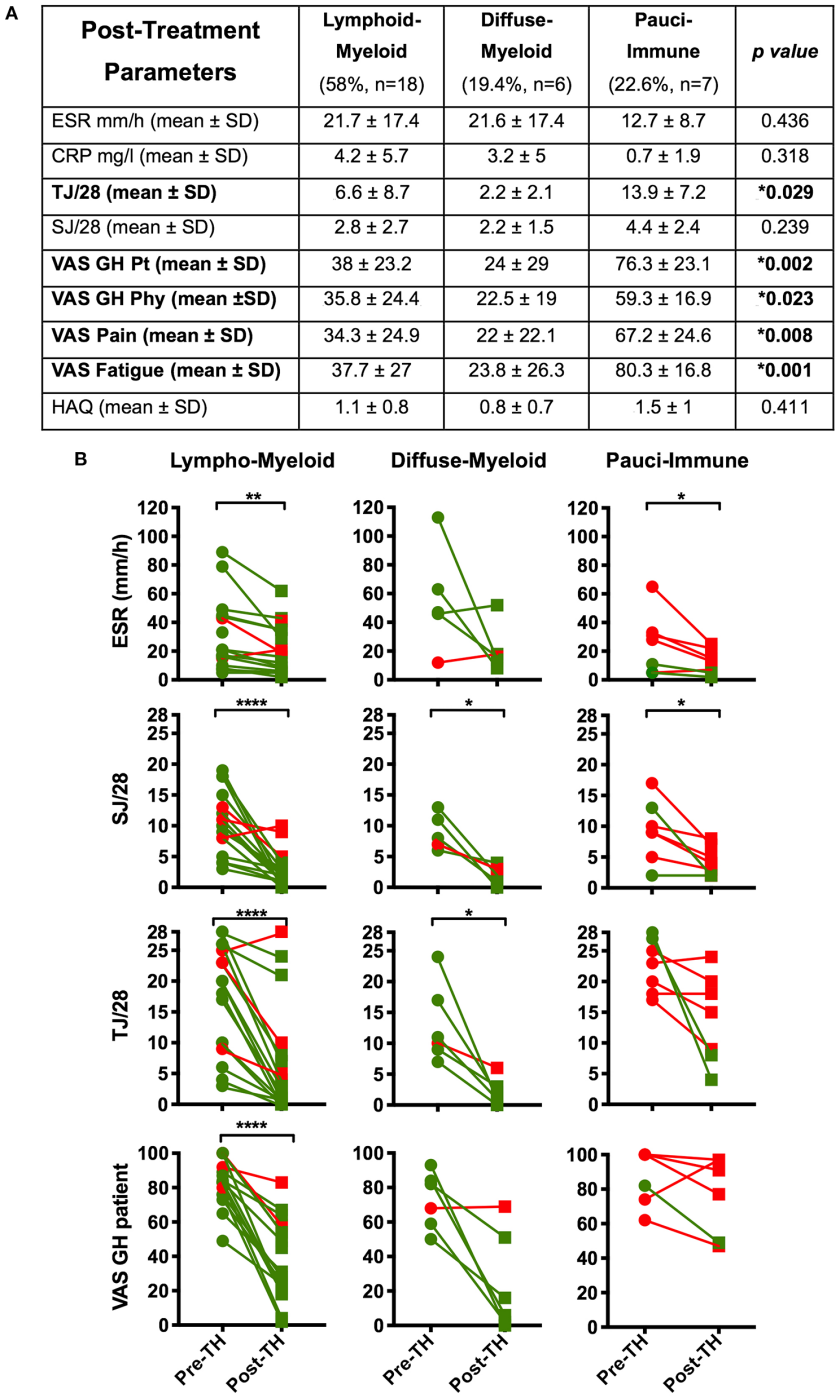
**(A)** Table summarizing the comparison by pathotype of the patients' clinical features at 12-weeks post-treatment (^*^Kruskal-Wallis with Dunn's correction). Significantly different variables are in bold. **(B)** Comparison of pre- (pre-TH) and post-treatment (post-TH) individual parameters of the DAS28 by pathotype. p values were calculated with Wilcoxon matched-pairs rank test (31 patients); ^****^p < 0.0001, ^***^p < 0.001, ^**^p < 0.01, ^*^p < 0.05. Green dots and line: “responders”; red dots and lines: “non-responders”. SD, Standard Deviation; ESR, erythrocyte sedimentation rate; CRP, C- Reactive Protein; TJ, Tender Joints; SJ, Swollen Joints; VAS, Visual Analog Scale (0–100); GH, Global Health; pt, patient; phy, physician; tir, tiredness; HAQ, Health Assessment Questionnaire.

We demonstrated a significantly higher level of post-treatment tender joint count and VAS scores for pain, fatigue and global health in pauci-immune in comparison with lympho- and diffuse-myeloid patients (Figure 4A): conversely, there were no significant differences in the number of swollen joints, ESR and CRP at 12-weeks post-treatment between pathotype groups.

Furthermore, when evaluating changes in individual components of the DAS28 score between baseline and 12-weeks follow up we saw the most significant falls in ESR, tender joint count, swollen joint count and VAS global health in the lympho-myeloid group. In the pauci-immune group only ESR level and swollen joint count significantly decreased after treatment (Figure 4B). Overall, this data suggests that while anti-TNF therapy is highly effective in the “inflammatory” pathotypes, it is less effective at reducing joint tenderness, pain and overall health scores in the pauci-immune patients.

### Clinical Response at 12-Weeks Associates With a Reduction in the Number of Sublining Macrophages and Size of Synovial B Cells Aggregates

**Figure 5:**
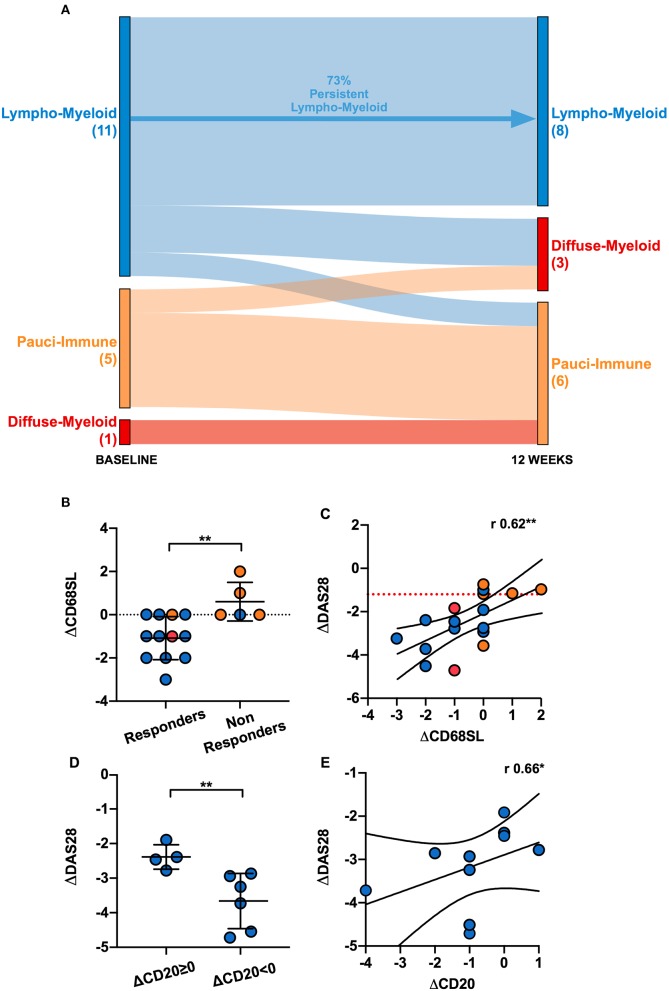
**(A)** Sankey diagram representing the shift of pathotypes from pre- to post-treatment of the 17 patients who had gradable tissue both at baseline and 12-weeks. **(B)** Comparison of the delta (Δ) CD68 sublining (SL) score between responders and non-responders. ΔCD68SL is the difference in the CD68SL semi-quantitative score (0–4) between post- and pre-treatment. Mean and standard deviation shown. ^**^p < 0.01 (Mann-Whitney). **(C)** Correlation plot showing ΔDAS28^[12−weeks−baseline]^ and ΔCD68SL^[12−weeks−baseline]^; r Spearman coefficient of correlation, ^**^p < 0.01. **(D)** Comparison of ΔDAS28 between patients with same/higher (ΔCD20≥0) or reduced CD20^+^ B-cells score (ΔCD20 < 0) post treatment. ^**^p < 0.01 (Mann-Whitney, mean and standard deviation shown). **(E)** Correlation plot showing ΔDAS28^[12−weeks−baseline]^ and ΔCD20^[12−weeks−baseline]^. r Spearman coefficient of correlation, ^**^p < 0.01. Only responder lympho-myeloid patients were analyzed in **(D,E)** (10 patients). **(B–E)** Individual dots are color-coded by pathotype (*blue*, lympho-myeloid; *red*, diffuse-myeloid; *orange*, pauci-immune).

In order to evaluate the changes in synovial histopathology between baseline and 12-weeks, we focused on a subset of 17 patients who had gradable synovial tissue at both time-points (Supplementary Table 1). At 12-weeks, of these 17 patients, 47% (8/17) were classified as lympho-myeloid, 17.7% (3/17) as diffuse-myeloid and 35.3% (6/17) as pauci-immune pathotype. Overall, 70.6% patients maintained the same pathotype as baseline, 23.5% changed to a less inflammatory and 5.9% to a more inflammatory histological patter. We then evaluated the change in synovial pathotype per patient between baseline and 12-weeks. We observed that, following anti-TNF treatment, the percentage of lympho-myeloid decreased from 58 to 47% while the number of pauci-immune patients increased from 22.6 to 35.3%. Furthermore, in the absence of B cell aggregates at baseline, patients did not develop B cells and/or plasma cells post-treatment, and so there was no transition to the most inflammatory pathotype (lympho-myeloid) at 12-weeks (Figure 5A).

Next, we assessed the change in CD68+ sublining macrophages between baseline and 12-weeks in responders and non-responders to certolizumab-pegol and observed a significant fall of this cell subset in responder patients (Figure 5B). We also confirmed a significant correlation between change in CD68+ sublining macrophages and change in DAS28 score between baseline and 12-weeks (Figure 5C). Similarly, we evaluated whether modulation of synovial CD20+ B cells at 12-weeks was related to clinical response. We analyzed patients with a lympho-myeloid pathotype at baseline who had a suitable synovial tissue for histological characterization post-treatment (11 patients). Only one patient was deemed as non-responder; therefore, in terms of clinical response, we did not detect any significant difference between patients with persistence (8/11, from baseline lympho-myeloid to lympho-myeloid at 12-weeks) or complete resolution of CD20+ B-cells aggregates (3/11, from baseline lympho-myeloid to diffuse-myeloid or pauci-immune at 12-weeks). We next stratified responders patients into those with a change in CD20 scores of ≥ 0 between baseline and 12-weeks, meaning same or higher B-cell score post-treatment, and those with a change of <0 (indicating a lower B-cell score at 12-weeks); then, we evaluated the mean change in DAS28 scores between the two groups. Our results suggested that patients with a reduction in the CD20+ B-cells score after treatment had a significantly greater improvement of the disease activity score at 12-weeks (Figure 5D). We also assessed the relationship between absolute change in DAS28 and differences in the CD20+ B-cell scores between baseline and 12-weeks and showed a significant correlation (*r* = 0.66, *p* < 0.05), suggesting that clinical improvement is associated with falls in CD20 scores and clinical worsening with increased scores (Figure 5E). These data demonstrate that depletion of synovial sublining macrophages and B cells are important factors in responsiveness to anti-TNF treatment.

## Discussion

Despite the considerable improvement in outcomes for RA patients since the introduction of TNFi therapy, clinicians are still unable to reliably identify patients most likely to respond to specific therapies, which remains a huge unmet clinical need. The identification of peripheral blood biomarkers of clinical response to TNFi therapy has been largely unsuccessful (3) and so focus has shifted to the examination of the primary site of inflammation in RA, i.e. synovial tissue (5). Early studies on this topic recognized the heterogeneity of rheumatoid synovitis and the presence of high- and low-inflammation synovial tissue, with the former being associated with higher disease activity and systemic markers of inflammation (18). More recently, numerous studies defining the cellular and molecular composition of the rheumatoid synovium both at single-cell (19, 20) and whole-tissue (6, 7) level have confirmed the heterogeneity and complexity of the tissue in its diseased state as well as its association with clinical phenotypes.

Here, we characterized for the first time the baseline features of the synovial tissue of a homogeneous group of 37 inadequate responders to csDMARDs by applying a histologic algorithm previously validated in a different and independent large cohort of early RA patients and evaluated whether synovial histological characteristics prior-to-treatment correlated with clinical response to the TNFα inhibitor certolizumab-pegol.

Firstly, we confirmed the presence of all three synovial pathotypes as described early in the disease and prior to any treatment intervention (6, 7). These include a pauci-immune pathotype, which, despite the absence of infiltrating inflammatory cells, can be detected in RA patients with active disease fulfilling UK NICE criteria for starting biologic treatment (DAS28 > 5.1). The relative prevalence of the lympho-myeloid pathotypes in this RA cohort with established, more longstanding disease was higher than in early, untreated RA [58% vs. 39% (6)]. This is in line with the recent observation in early RA that patients characterized by a lympho-myeloid pathotype at disease onset are more likely to progress in terms of increasing joint damage as assessed on x-ray and are subsequently more likely to require biologic therapy at 12 months follow up (8).

The relationship between the baseline histological characteristics and the clinical response was assessed at 12-weeks by the change in the DAS28 pre- and post-treatment, since improvement in DAS28 <1.2 at 12-weeks has been shown to be the best predictor of inadequate response at 1 year (21, 22). Overall, 67.6% of the treated patients improved their baseline DAS28 of ≥1.2 and were deemed as responders, aligned with the standard rates of response to TNFα inhibitors in clinical trials (23). However, when patients were categorized according to the baseline histological pattern, the rate of responders enriched from 67.6% up to 83.3% in both the lympho-myeloid and diffuse-myeloid pathotypes; conversely, a pauci-immune synovitis, i.e. scanty synovial inflammatory infiltrate, associated with a significantly lower rate of response (28.6%), impaired reduction of the DAS28 from pre- to post-treatment and higher absolute values of DAS28 at 12-weeks. Notably, and in line with these results, a pauci-immune-fibroblast signature in synovium of treatment-naïve patients (independent from the cohort included in this manuscript) has been found to be associated with resistance to csDMARDs in early-RA (7).

Moreover, by dissecting the post-treatment DAS28 into its individual variables, we demonstrated that the main drivers of the inadequate response in the pauci-immune patients at 12-weeks were the high number of tender joints and the patient global health VAS score whereas ESR and swollen joint count were comparable among the three pathotypes. All the patient-reported-outcomes recorded at 12-weeks, including fatigue and pain, were consistently higher in pauci-immune patients. Thus, although anti-TNF therapy reduced ESR and CRP in pauci-immune patients, it was less effective at reducing joint tenderness and pain scores in pauci-immune patients compared to the other pathotypes. Interestingly, the dissociation of pain scores from markers of inflammation has been recently described in a group of patients bearing a molecularly-defined “low inflammatory” subtype of synovitis characterized by the up-regulation of neurogenesis pathways and TGF-β (24). Thus, altogether, these data support the notion of a distinct “hyperalgesic” clinical phenotype linked to the pauci-immune pathotype. The role played by the synovial histopathology in predicting the response to TNFi has been long debated, particularly, because of the discordant findings coming from various observational studies. To date, chief attention has been given to lymphocytic structures within the synovial tissue. Their presence has been associated with the achievement of clinical response in some (25), though, not all studies (26); alternatively, it has been proposed that the disruption of the lymphoid follicles is instead critical for the success of the treatment (26), hence leaving the question substantially unanswered. Similarly, molecular analysis of the synovial tissue showed that, in some cases, an up-regulation of inflammation-related and myeloid genes characterized responder patients (11, 12) whereas, in others, high synovial content of pro-inflammatory IL7-receptor and IL-18 predicted the absence of response to TNFα blockade (27).

Here, our results suggest that both the baseline high-inflammatory pathotypes lympho-myeloid and diffuse-myeloid, differentiated by the presence/absence of B and/or plasma cell aggregates but both sharing a substantial infiltration by macrophages, had significantly better chances to respond to TNFα inhibition by certolizumab-pegol than patients with a pauci-immune pathotype. Our findings are in line with previous data demonstrating that a myeloid gene signature (12) as well as higher levels of synovial TNFα (28) pre-treatment predict enhanced response to TNFα-blockade. Conversely, the virtual absence of immune cells seems favoring the absence of clinical response, which may be potentially driven by other pathways including maladaptive pain response.

Finding that clinical response to certolizumab-pegol is significantly associated with a fall in CD68+ sublining macrophages is in line with the well-established literature recognizing the decrease in synovial macrophages as a validated biomarker of the clinical efficacy of various therapeutic interventions (29, 30). Several studies involving the use of anti-TNFα agents, including our previous observations (31), have already shown that the reduction in the number of sublining macrophages from baseline, also at early time-points after starting the treatment, associated with clinical response in RA (32, 33); we have here further confirmed this evidence. This early reduction in sublining CD68-positive cells in future responders to TNFi has also been mirrored in the peripheral blood by the change in myeloid-related-protein (MRP) 8/14 (34).

We also showed that if B cells and plasma cells are absent at baseline, as occurs in the diffuse-myeloid and pauci-immune pathotypes, there is no evolution toward a lympho-myeloid pathotype after treatment, in agreement with early works demonstrating a reduced synovial cellular infiltration upon treatment with infliximab (35). We observed that a decrease in the CD20^+^ B-cell score post-treatment associated with improvement in the DAS28. Thus, depletion of synovial sublining macrophages and B cells are important factors for effective response to anti-TNF treatment. Overall, these data suggest that a shift toward a less inflammatory synovial environment after treatment associates with greater improvement in clinical disease activity, in line with similar observations in early RA patients following csDMARD therapy (7). Because certolizumab-pegol exerts its action by dampening inflammation through inhibition of TNFα, it is reasonable that it does not adequately target the pauci-immune pathotype. Hence, the development of novel therapeutics targeting the fibroblastic synovial component and aberrant nociceptive pathways may be required in the pauci-immune patient cohort and support the notion that a personalized medicine, biomarker driven approach to treating patients with arthritis is required.

In conclusion, we have demonstrated that the analysis of the synovial histopathology may be a valuable tool to discern among clinically indistinguishable patients those with less chance of responding to TNFα-blockade, and additional research efforts are required to properly target the pauci-immune pathotype.

## Data Availability Statement

The datasets generated for this study are available on request to the corresponding author.

## Ethics Statement

The studies involving human participants were reviewed and approved by Rec. No. 10/H0801/47 (NRES Committee London Central). The patients/participants provided their written informed consent to participate in this study.

## Author Contributions

All authors have contributed to different degrees to patient recruitment, data generation, data analysis, and writing and/or revising the manuscript.

## Conflict of Interest

The authors declare that the research was conducted in the absence of any commercial or financial relationships that could be construed as a potential conflict of interest.
