# Supplementary figures and images for: A Pauci-Immune Synovial Pathotype Predicts Inadequate Response to TNFα-Blockade in Rheumatoid Arthritis Patients
Source: Front Immunol. 2020 May 5;11:845. doi: 10.3389/fimmu.2020.00845 (PMC7214807; doi:10.3389/fimmu.2020.00845)

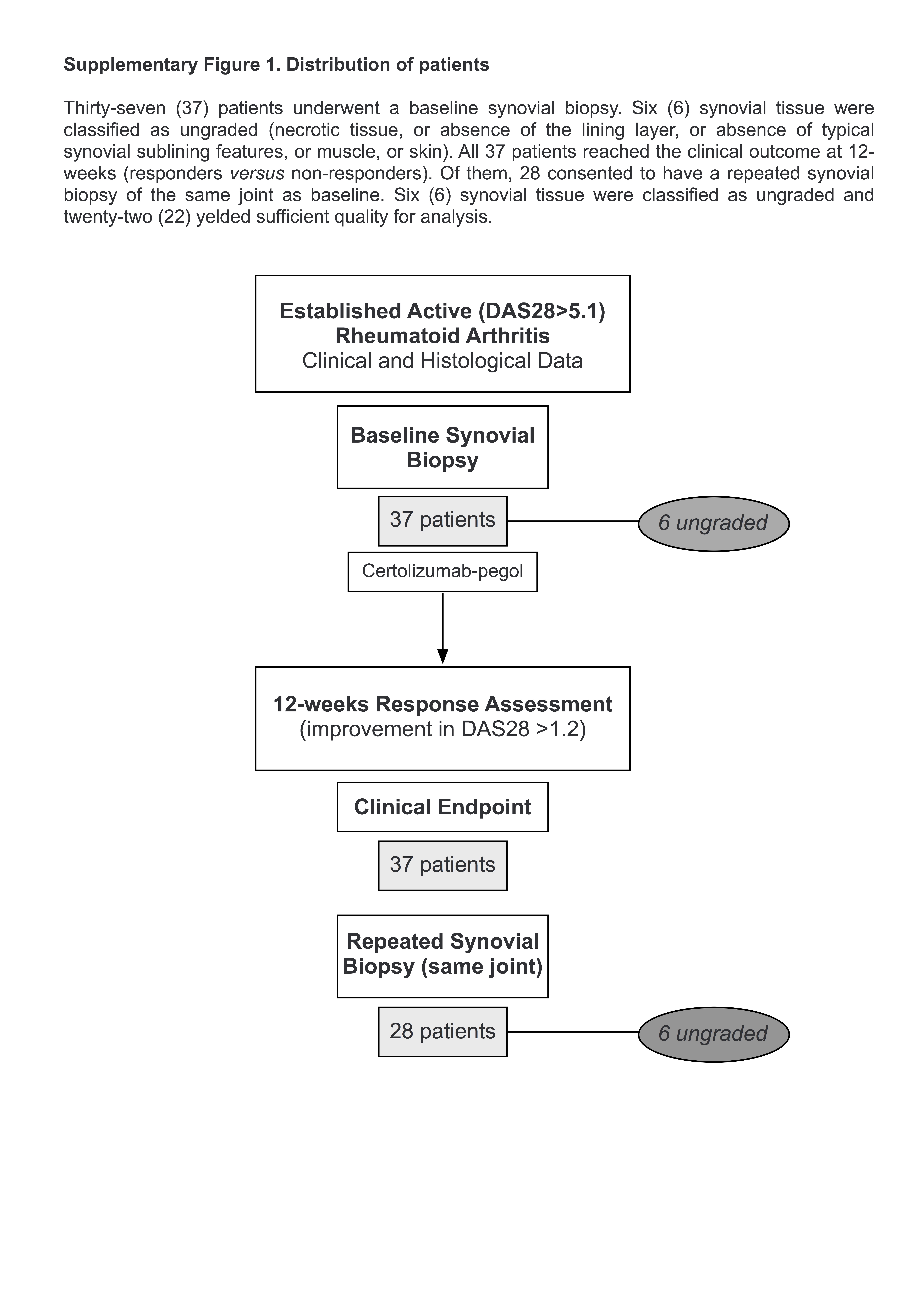

Supplement: Supplementary file 1 [file Image_1.JPEG]

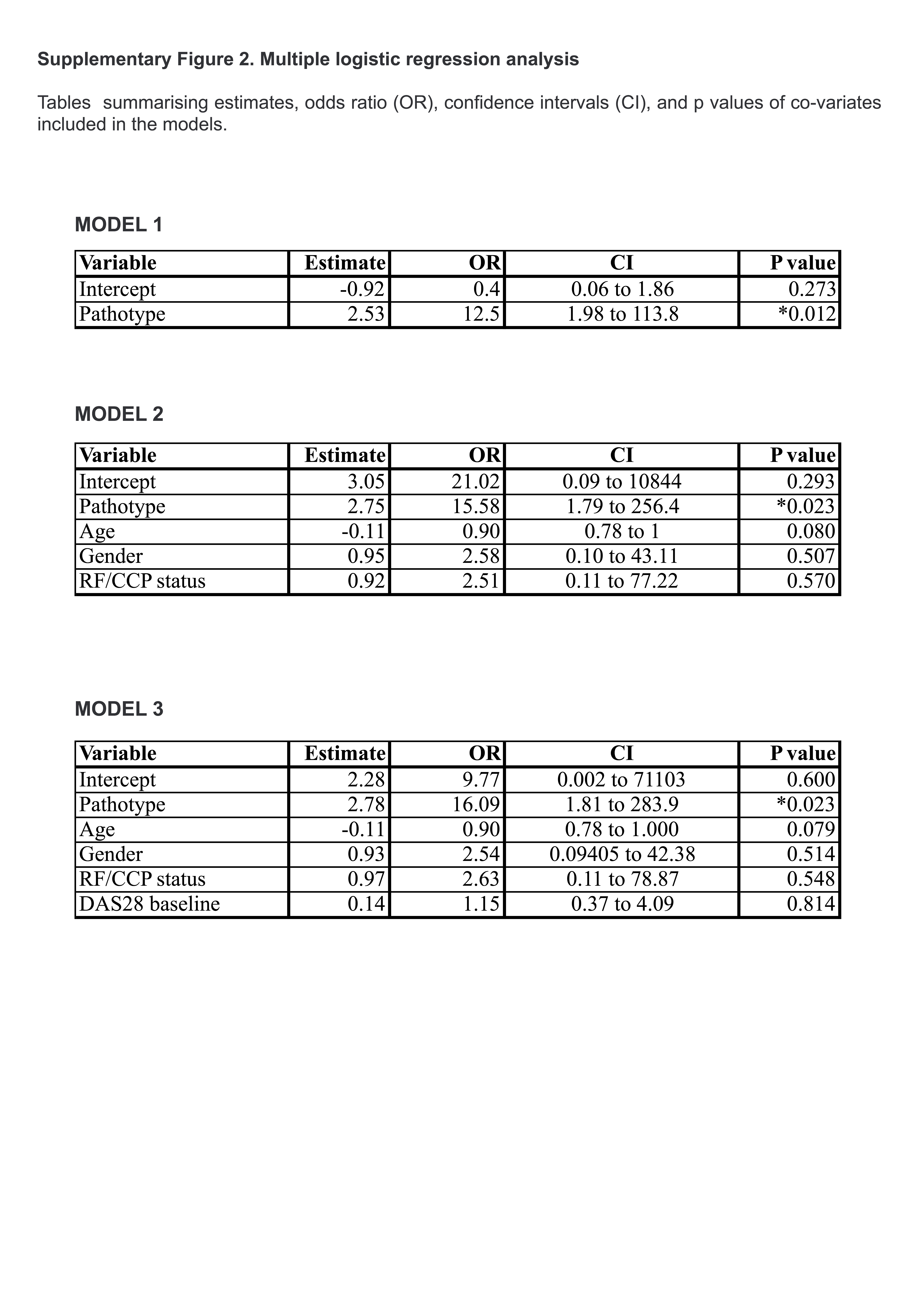

Supplement: Supplementary file 2 [file Image_2.JPEG]

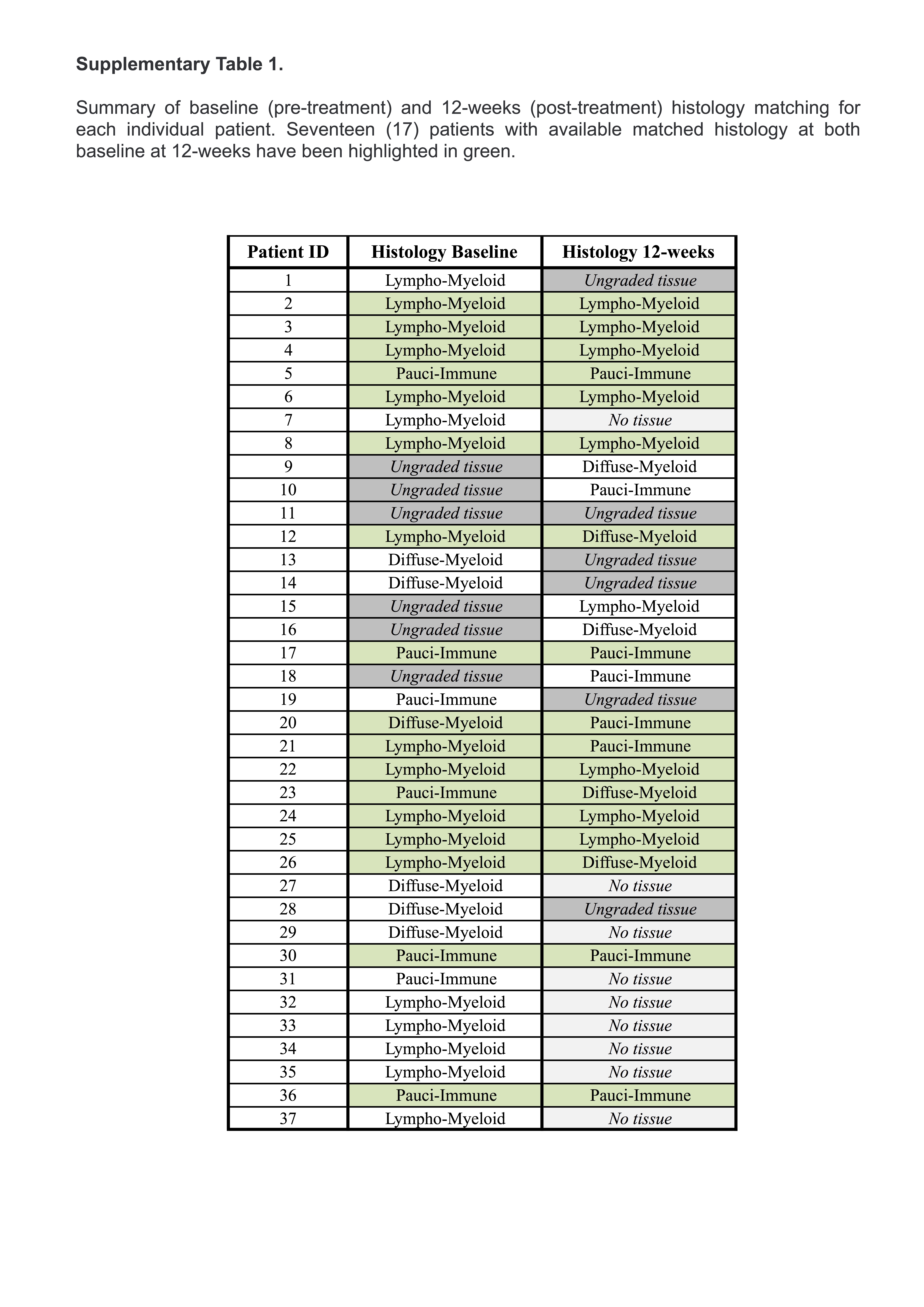

Supplement: Supplementary file 3 [file Image_3.JPEG]

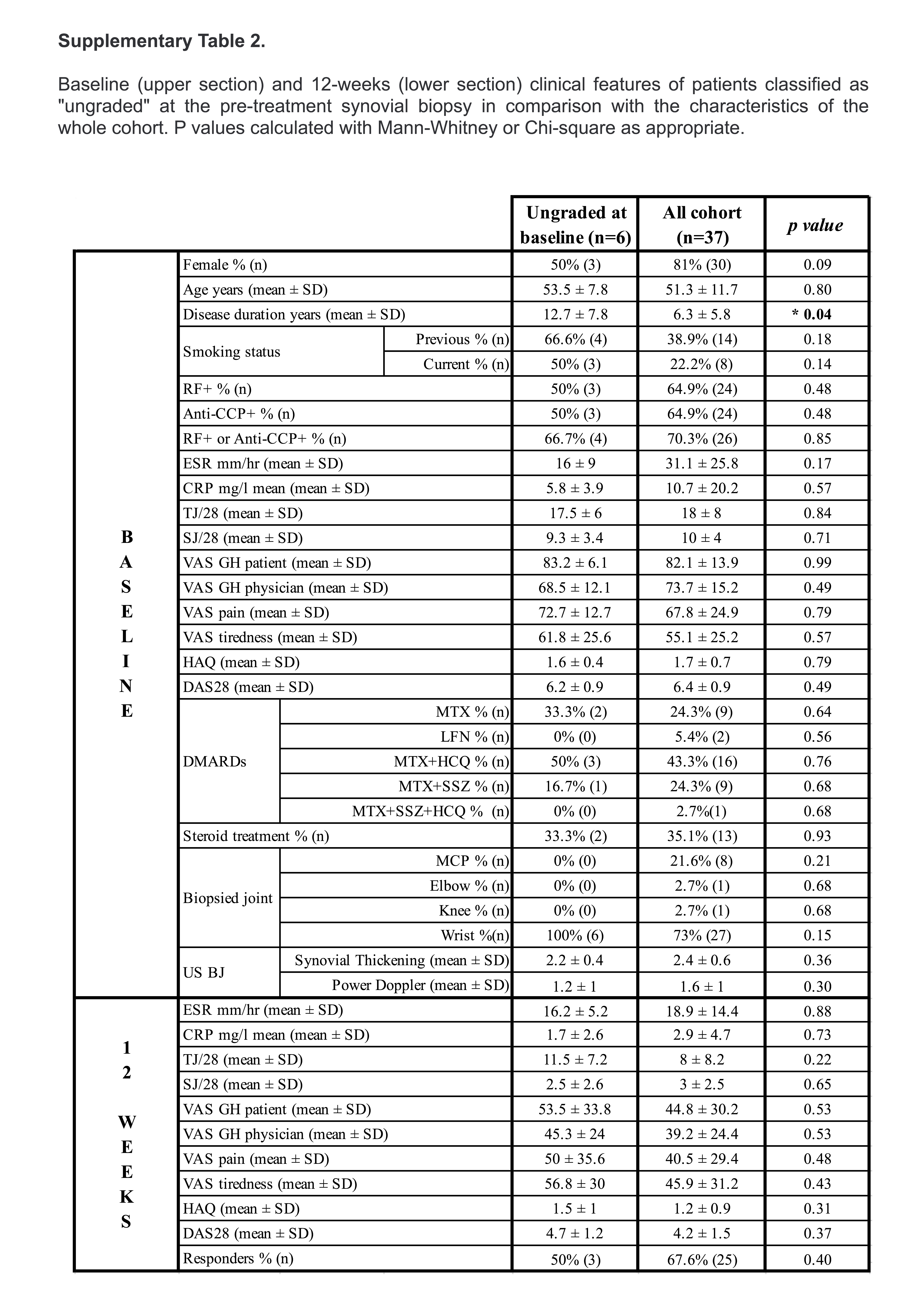

Supplement: Supplementary file 4 [file Image_4.JPEG]

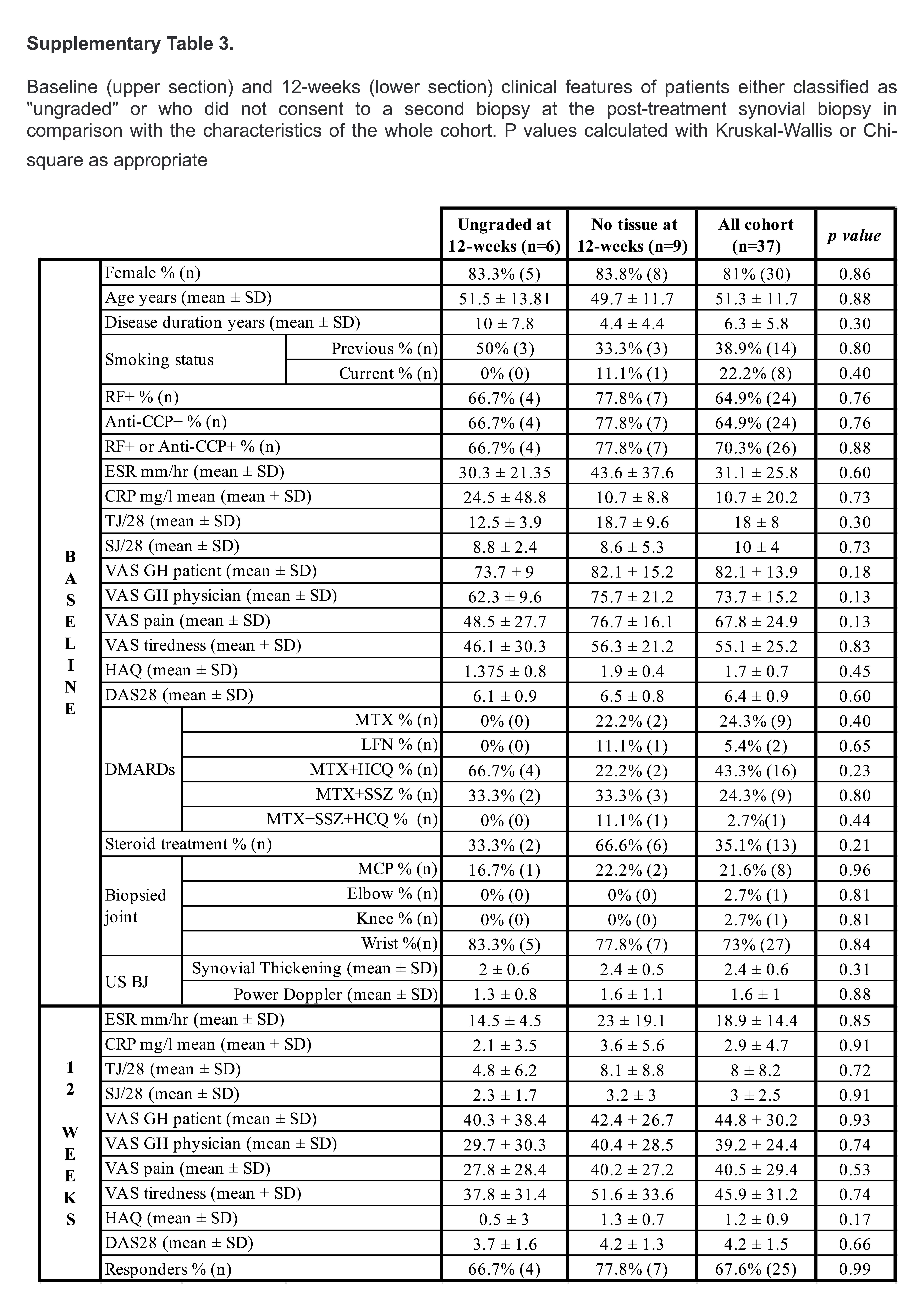

Supplement: Supplementary file 5 [file Image_5.JPEG]
